# The risk of endocrine interventions in carriers of a genetic predisposition for breast and gynecologic cancers: recommendations of the German Consortium for Hereditary Breast and Ovarian Cancer

**DOI:** 10.1007/s00432-024-05936-7

**Published:** 2024-09-11

**Authors:** O. Ortmann, S. Schüler-Toprak, K. Kast, For The working group “Hormones”, The German Consortium for Hereditary Breast, Ovarian Cancer (GC-HBOC), O. Ortmann, S. Schüler-Toprak, K. Kast, T. Fehm, A. Hahne, D. Huber, E. Kühnle, K. Mohr, K. Rhiem, S. Seitz, D. Speiser

**Affiliations:** 1 Department of Gynecology and Obstetrics, University Medical Center Regensburg, Landshuter Str. 65, 93055 Regensburg, Germany; 2 Center for Familial Breast and Ovarian Cancer and Center for Integrated Oncology, Medical Faculty, University Hospital Cologne, Cologne, Germany

**Keywords:** Oral contraception, Fertility treatment, Hormone therapy, *BRCA1* and *BRCA2*, Breast cancer, Ovarian cancer

## Abstract

**Purpose:**

To support doctors in counselling women with genetic predisposition for breast or gynecologic cancers on endocrine interventions.

**Methods:**

Evidence on the safety of endocrine interventions for fertility treatment, contraception, hormone replacement therapy after risk-reducing salpingo-oophorectomy (RRSO) or treatment of symptoms during peri- and postmenopause was analysed for carriers of probably pathogenic and pathogenic variants in *BRCA1* or *BRCA2* (*BRCA1/2*-pV), in other breast and ovarian cancer genes and the Lynch Syndrome. Cancer risks were compared with data on risks for the general population.

**Results:**

Data on risk modulation of endocrine interventions in women with genetic predisposition is limited. Ovarian hyperstimulation for fertility treatment may be performed. Oral contraceptives should not be used to reduce ovarian cancer risk in *BRCA1/2*-pV carriers. Premenopausal *BRCA1/2*-pV carriers and carriers of pV in Lynch Syndrome genes should be offered hormone replacement therapy (HRT) after RRSO, to prevent diseases caused by estrogen deficiency.

**Conclusion:**

Effect direction and strength of risk modulation by endocrine interventions is similar to the general population. Participation of individuals at risk in prospective registries is recommended.

## Introduction

Women with a genetic predisposition for breast or gynecological cancers consider the use of endocrine interventions for fertility treatment, contraception, hormone replacement therapy (HRT) after risk-reducing salpingo-oophorectomy (RRSO) or treatment of symptoms during peri- and postmenopause. They are concerned about an increase in their already genetically determined cancer risk. Statements and recommendations contained in this consensus paper intend to support doctors in counseling these women.

Non-genetic risk factors influence the penetrance of genetic predisposition to breast and ovarian cancer. Even if a disease-relevant variant (probably pathogenic, class 4 or clearly pathogenic variant (pV), class 5) is detected in one of the breast and/or ovarian cancer genes, the lifetime probability of developing the disease is less than 100%. For breast cancer risk, a distinction is made between genes with a high lifetime breast cancer risk (> 50%) and those with a moderate breast cancer risk (approx. 20–40%).

Non-genetic risk factors together with other genetic factors, e.g. the polygenic risk score (PRS), can also determine the level of risk and age of onset. There is currently sufficient evidence showing that factors that influence risk for sporadic breast and ovarian cancer are also of clinical significance in cases of genetic predisposition. However, studies on carriers of genes other than *BRCA1* and *BRCA2* are not available.

The absolute risks are also largely unclear, in particular the age-dependent risks of age groups that are poorly recorded in the registry studies such as young female carriers of a probably pathogenic or pathogenic variant in *BRCA1* or BRCA2 genes (*BRCA1/2*-pV) before the age of 25 and those after the age of 70, as well as male carriers. In addition, the data situation for female BRCA2-pV carriers is less well-founded than for *BRCA1*. For example, two thirds of the *BRCA1/2*-pV documented in the HerediCaRe database of the GC-HBOC are in the *BRCA1* gene and the proportion of female carriers of *BRCA1*-pV is also higher in international European study groups.

Non-genetic risk factors include endogenous factors such as the timing of menarche or breast density. However, they also include endocrine interventions, such as ovarian hyperstimulation for fertility treatment, hormonal contraception, HRT after RRSO, or menopausal hormone therapy. Sensitive areas of quality of life and long-term health are affected, so that the omission of the intervention itself can represent a harm and this must be weighed against any risks.

Recommendations for oncologically healthy (non-diseased) carriers may differ from oncologically affected (diseased) women. Whether an endocrine intervention causes risk for cancer, relapse or distant metastasis also depends on whether the type of cancer (breast, ovary, endometrium, colon, etc.) is considered hormone-sensitive. Therefore, where evidence is available, a distinction is made in the chapters listed between *BRCA1/2*-pV carriers and carriers of pV in another breast and/or ovarian cancer risk gene and those with pV in the Lynch genes. Since evidence on the effect of endocrine interventions on cancer risk in carriers of a genetic disposition is limited, the chapters include the state of knowledge on the effects in the general population. Although the transferability is not formally correct, this appears to be helpful in decision-making, as there is limited evidence regarding different impacts of the presented interventions for *BRCA1/2*-pV carriers and the general population.

## Methods

A comprehensive literature search was conducted using the PubMed database. The objective was to identify relevant articles focusing on endocrine interventions in various contexts, specifically for women carrying pathogenic variants in *BRCA1* or *BRCA2*, other risk genes for breast and/or ovarian cancer and Lynch syndrome. Articles on endocrine interventions during fertility treatment, contraception, hormone replacement therapy (HRT) following risk-reducing salpingo-oophorectomy (RRSO) and hormonal treatment of symptoms during peri- and postmenopause were of particular interest. The search strategy employed a combination of keywords and MeSH (Medical Subject Headings) terms to ensure a comprehensive retrieval of relevant articles. The following keywords and phrases were used, individually and in combination, to perform the search: “endocrine interventions”, „fertility treatment”, „contraception”, “hormonal replacement therapy” OR “HRT”, “risk-reducing salpingo-oophorectomy” OR “RRSO”, “perimenopause” OR “postmenopause”, “BRCA1” OR “BRCA2”, “Breast cancer risk genes”, “Ovarian cancer risk genes”, “Lynch syndrome”, “breast cancer”, “ovarian cancer”, “endometrial cancer”, “estrogens”, “progestins”. The search was limited to articles published in English. No restrictions were placed on the publication date to include a comprehensive range of studies. To compare the data with studies, statements, and recommendations for the general population, the following current German S3-guidelines were referenced: “Peri- and postmenopause—diagnosis and interventions”, “Screening, diagnosis, treatment and follow-up of breast cancer”, “Diagnosis, treatment and follow-up of malignant ovarian tumors”, “endometrial cancer” and “Hormonal contraception”. These guidelines provided a benchmark for evaluating the specific risks and recommendations for women with genetic predispositions against the general population standards.

## Ovarian stimulation for fertility treatment

Does ovarian stimulation for fertility treatment influence the risk for breast or ovarian cancer of non-diseased BRCA1/2-pV carriers?

Statements:

- Ovarian stimulation with clomiphene or gonadotropins does not seem to increase the risk of breast cancer in *BRCA1/2*-pV carriers.
- Due to the limited number of studies and their methodological weaknesses, definitive statements on oncological safety are not possible.
- Available data do not allow any clear statement on a possible risk-increasing effect for ovarian cancer.

Recommendations:

- Ovarian hyperstimulation may be performed for fertility treatment in *BRCA1/2*-pV carriers.
- Limited evidence on oncological safety should be explained to patients.

Does ovarian stimulation for fertility treatment influence the risk for breast or ovarian cancer of non-diseased carriers of pV in breast and/or ovarian cancer genes others than *BRCA1* or *BRCA2*?

Statements:

- Ovarian hyperstimulation with clomiphene or gonadotropins is unlikely to increase breast cancer risk in carriers of a pathogenic variant (pV) in breast cancer genes other than *BRCA1* or *BRCA2*.
- Based on the findings in *BRCA1/2*-pV carriers, available data do not allow any clear statement to be made about a possible risk-increasing effect on the risk of ovarian cancer in carriers of a pV in other breast and/or ovarian cancer risk genes.

Recommendations:

- Ovarian hyperstimulation may be performed for fertility treatment in carriers of pV in breast and/or ovarian cancer risk genes other than *BRCA1* or *BRCA2*.
- Limited evidence on oncological safety should be explained to patients.

### Cancer risk due to ovarian stimulation for fertility treatment in the general population

There are extensive case–control and cohort studies that have investigated the use of ovarian stimulation drugs on the risk of borderline ovarian tumors and ovarian cancer. Studies conducted in the early 1990s showed an increase in risk. However, it must be taken into account that both prospective and retrospective observational studies can produce a false positive result due to surveillance bias. For example, transvaginal sonographies are performed more frequently in women undergoing fertility treatment. In the largest study published up to 2009 with over 50,000 women, only nulliparity was a relevant risk factor for the development of ovarian cancer. None of the drugs used to induce ovulation were associated with an increased risk of ovarian cancer. However, it must be noted that the mean age of women was 30 years at the initial evaluation and was only 47 years after follow-up. Therefore, an effect on the risk of ovarian cancer cannot be ruled out with certainty for longer observation periods (Jensen et al. 2009).

Only few studies have been conducted on the relationship between ovulation induction and the risk of endometrial cancer. In a metaanalysis in which 110,000 women were treated with in-vitro fertilization (IVF), no significant associations were found between IVF, ovarian and endometrial cancer, if infertile women served as a comparison group (Siristatidis et al. 2013). In contrast, a smaller study from Israel reported an increased risk for endometrial and ovarian cancer, but not breast or cervical cancer, when comparing women with (n = 4363) and without fertility treatment (n = 101,668) (Kessous et al. 2016).

Studies on the risk of breast cancer revealed contradictory results with slight increases and decreases in risk. A comprehensive metaanalysis that included over 1.5 million women found no significant difference in treated women compared to the general population or infertile women (Sergentanis et al. 2014). A Dutch study also showed no increase in the risk of breast cancer as a result of fertility treatment compared to the standardized incidence ratio (SIR) of the Dutch population (n = 25,108; median follow-up 21.4 years), even with a longer follow-up (van den Belt-Dusebout et al. 2016).

A recent systematic review with metaanalysis identified a total of 228 studies in which the association between fertility treatments and the risk of breast, ovarian, endometrial or cervical cancer was investigated. The incidences of breast and endometrial cancer were not significantly different between treated and non-treated women. The overall analysis of ovarian cancer incidence also revealed no significant differences between the two groups. However, there was a significant increase in the risk of borderline tumors (odds ratio (OR) 1.69). Subgroup analyses showed that the incidence of ovarian cancer was significantly higher in women treated with IVF and clomiphene (OR 1.32 and OR 1.40 respectively). In contrast, the incidence of breast and cervical cancer was significantly lower in the IVF-treated subgroup compared to the non-treated group (OR 0.75 and OR 0.58, respectively). There was no increase in the overall cancer risk (Barcroft et al. 2021).

### Influence of ovarian stimulation for fertility treatment on the risk of ovarian cancer in non-diseased *BRCA1/2*-pV carriers

Data on the risk of ovarian cancer after fertility treatment in *BRCA1/2*-pV carriers is limited (Huber et al. 2020a). Two retrospective studies are available. In an Israeli cohort study, 1052 *BRCA1/2*-pV carriers were included, 164 of whom received fertility treatment or medication for ovarian stimulation (Perri et al. 2015). In a case–control study, 1882 *BRCA1/2*-pV carriers were included, of whom 941 belonged to the case group with a history of ovarian cancer (Gronwald et al. 2016). In only 64 of the included carriers, infertility treatment was performed or stimulation medication was administered. Both studies showed no association between fertility treatment and the risk of ovarian cancer.

A Cochrane Review, which included data from 13 case–control studies and 24 cohort studies, also of *BRCA1/2*-pV carriers, described a possible increase in the risk of ovarian cancer and borderline tumors after fertility treatment in subfertile women (Rizzuto et al. 2019). However, the significance of the review is weakened by the small number of cancer cases observed and the presence of confounding factors that increase the risk of ovarian cancer.

### Influence of ovarian stimulation for fertility treatment on the risk of breast cancer in non-diseased *BRCA1/2*-pV carriers

Available data regarding the risk of breast cancer after fertility treatment is likewise limited (Huber et al. 2020a). In a case–control study from 2008, in which 2760 *BRCA1/2*-pV carriers were included, a possible risk-increasing effect of IVF treatment was observed and an unfavorable effect of gonadotropins was described (Kotsopoulos et al. 2008). However, this was not statistically significant (OR 2.32; 95% CI 0.91–5.95; p = 0.08) and must be regarded with caution due to the small number of cases (n = 10 vs. 16, controls vs. cases with breast cancer) (Kotsopoulos et al. 2008). Instead, the study by Perri et al. 2021 suggested a non-significant protective effect of treatment with gonadotropins (HR 0.54; 95% CI 0.28–1.01; p = 0.06) with a higher albeit small number of cases (92 vs 27, controls vs. cases with breast cancer) (Perri et al. 2021).

A recent systematic review with metaanalysis from 2022 included five cohort studies and three case–control studies—including the two already mentioned—that investigated the association between fertility treatment and the incidence of breast cancer in women with pV in *BRCA1* or *BRCA2* genes (Liu et al. 2022). They found no significant increase in the risk of breast cancer due to fertility treatment in *BRCA1/2*-pV carriers (pooled OR 1.02, 95% CI 0.74–1.4) (Liu et al. 2022). Even after distinguishing between pV in *BRCA1* and *BRCA2* genes, there was no increased risk of breast cancer for the subgroups (pooled OR for *BRCA1* 1.18, 95% CI 0.81–1.72; pooled OR for *BRCA2* 0.54, 95% CI 0.09–3.34) (Liu et al. 2022). Furthermore, it was investigated to what extent different fertility treatment methods could have a different influence on the risk of breast cancer in *BRCA1/2*-pV carriers. Neither IVF (pooled OR 0.75, 95% CI 0.51–1.1), stimulation with clomiphene (pooled OR 1.07, 95% CI 0.78–1.45) nor gonadotropins (pooled OR 1.32, 95% CI 0.8–2.18) showed an increased risk of breast cancer in *BRCA1/2*-pV carriers (Liu et al. 2022).

### Influence of ovarian stimulation for fertility treatment on cancer risk in diseased carriers of a genetic predisposition for breast, ovarian and endometrial cancer

Patients can attempt pregnancy after treatment for early breast cancer (S3-Guideline “Screening, Diagnosis, Treatment and Follow-up of Breast Cancer”). They should be informed about fertility-preserving measures before initiating treatment.

A prospective cohort study from 2016 examined breast cancer patients who had undergone ovarian stimulation treatment with letrozole and gonadotropins as part of fertility protection prior to planned chemotherapy (Kim et al. 2016). In 47 of the patients, pV in *BRCA1* or *BRCA2* genes were known. There was no effect of the stimulation treatment on overall and disease-free survival in patients with and without a genetic predisposition. Data on the cancer risk associated with hormonal stimulation treatment in carriers of pV in breast and ovarian cancer genes other than *BRCA1* and *BRCA2* are not available. Overall, there is no evidence supporting a deviation from these recommendations when counseling patients with a genetic predisposition to breast cancer.

Patients of reproductive age who have endometrial or ovarian cancer should be informed about fertility-preserving therapies (S3-Guideline “Endometrial Cancer”; S3-Guideline “Diagnosis, Treatment and Follow-up of malignant Ovarian Tumors”). There is no evidence supporting a deviation from these recommendations when counseling patients with a genetic predisposition for ovarian or endometrial cancer.

## Hormonal contraceptives

Does the use of hormonal contraceptives influence breast or ovarian cancer risk of non-diseased *BRCA1/2*-pV carriers?

Statements:

- Oral contraceptives lead to a significant reduction of ovarian cancer risk in *BRCA1/2*-pV carriers.
- There is evidence of an age-dependent, risk-increasing effect of oral contraceptives on breast cancer risk in *BRCA1/2*-pV carriers.

Recommendations:

- Oral contraceptives should not be used to reduce ovarian cancer risk in *BRCA1/2*-pV carriers.
- Due to the possible increase in the risk of breast cancer in users of oral contraceptives, *BRCA1/2*-pV carriers should only use them taking age into account and after careful consideration.

Does the use of hormonal contraceptives influence breast or ovarian cancer risk of non-diseased carriers of pV in other risk genes for breast and/or ovarian cancer?

Statements:

- Oral contraceptives probably lead to a significant reduction of ovarian cancer risk in carriers of pV in breast and/or ovarian cancer risk genes other than *BRCA1* or *BRCA2*.
- There is probably an age-dependent, risk-increasing effect of oral contraceptives on breast cancer risk in carriers of pV in breast and/or ovarian cancer risk genes other than *BRCA1* or *BRCA2*.

Recommendations:

- Oral contraceptives should not be used to reduce ovarian cancer risk in carriers of pV in breast cancer risk genes other than *BRCA1* or *BRCA2*.
- Due to the possible increase in the risk of breast cancer following the use of oral contraceptives, carriers of pV in breast cancer risk genes other than *BRCA1* or *BRCA2* should only use them taking age into account and after careful consideration.

### Ovarian cancer risk due to the use of hormonal contraceptives in the female general population

In the general population, the risk-reducing effect of hormonal contraceptives on ovarian cancer risk is considered certain. In 2008, a pooled analysis of 45 epidemiological studies showed a relative risk reduction by a factor of 0.73 (95% CI 0.70–0.76, p < 0.0001) with a longlasting effect when taking oral contraception (Beral et al. 2008). The strength of this protective effect was dependent on the duration of use of oral contraception and was in some cases still detectable up to 30 years after discontinuation of contraception. Further studies have confirmed this effect for the use of other hormonal contraceptives (Lurie et al. 2008; Moorman et al. 2008; Hannaford et al. 2010). There is also evidence of a reduced risk of ovarian cancer by the use of a progestin-releasing intrauterine device (Soini et al. 2016).

### Breast cancer risk due to the use of hormonal contraceptives in the female general population

The influence of hormonal contraceptives on the risk of breast cancer in the general population has not been conclusively clarified (S3-Guideline “Hormonal Contraception”). As early as 1996, a pooled re-analysis of 54 epidemiological studies showed a relative increase in breast cancer risk by a factor of 1.24 while taking oral combined contraceptives. This is no longer detectable 10 years after discontinuation of oral contraception (Collaborative Group on Hormonal Factors in Breast Cancer 1996). Overall, it is assumed that the various forms of hormonal contraception slightly increase the risk of disease, although the level of evidence is low (Cibula et al. 2010). There are indications that oral and non-oral progestin-only contraceptives, e.g. progestin-releasing intrauterine devices (IUD), also have a comparable risk-increasing effect (Mørch et al. 2017; Fitzpatrick et al. 2023).

### Influence of the use of hormonal contraceptives on ovarian cancer risk of non-diseased *BRCA1/2*-pV carriers

A reduction in ovarian cancer risk following the use of oral contraceptives has also been observed for carriers of pV in *BRCA1* or *BRCA2* genes in several studies (Huber et al. 2020b). A metaanalysis from 2013 in which 4,363 carriers of pV in *BRCA1* or *BRCA2* genes were included, showed a significant risk reduction with an OR of 0.58 (95% CI 0.46–0.73) (Moorman et al. 2013). Another metaanalysis from 2023 with more than 10,000 carriers of pV in *BRCA1* or *BRCA2* genes confirmed this and described a greater risk reduction with longer duration of use (van Bommel et al. 2023). This metaanalysis included a retrospective cohort study published in 2021, which included 3989 carriers of pV in the *BRCA1* gene and **2445** in the *BRCA2* gene (Schrijver et al. 2021). The study found a significant risk reduction for carriers of pV in the *BRCA1* gene (HR 0.51; 95% CI 0.36–0.71) and a nonsignificant risk reduction for *BRCA2*-pV carriers (HR 0.65; 95% CI 0.35–1.19) (Schrijver et al. 2021). In multivariate analyses, it was shown that the risk was significantly reduced with 5–9 years of use compared to less than 5 years of use (HR 0.67; 95% CI 0.40–1.12). With over 10 years of use, the HR was 0.37 (95% CI 0.19–0.73) (p = 0.008). After discontinuation of therapy, the risk reduction persisted over 15 years (Schrijver et al. 2021). In 2022, the Hereditary Ovarian Cancer Clinical Study Group also showed in a case–control study with 1,733 matched couples that the use of an oral contraceptive in *BRCA1/2*-pV carriers leads to a significantly reduced risk of ovarian cancer (OR 0.59; 95% CI 0.49–0.71) (Xia et al. 2022). There was also initial evidence of risk reduction by the use of contraceptive implants and injectable hormonal contraceptives (Xia et al. 2022).

In the future, it will be possible to take the expected risk reduction into account when estimating the individual ovarian cancer risk using a model such as CanRisk. Even if risk-reducing salpingo-oophorectomy (RRSO) could not be completely avoided for the high-risk genes *BRCA1* and *BRCA2*, for example, it is conceivable that the risk reduction could shift the timing to a later age. This is currently subject of further research. Timely RRSO at the age of 35/40 (*BRCA1*/*BRCA2*) is still recommended, provided that family planning has been completed.

### Influence of the use of hormonal contraceptives on breast cancer risk of non-diseased *BRCA1/2*-pV carriers

With regard to breast cancer risk after use of oral contraceptives in *BRCA1/2*-pV carriers, data are heterogeneous (Huber et al. 2020b). As discussed by Cibula et al., some older retrospective studies show an increase in the risk of breast cancer after taking oral contraceptives in *BRCA1/2*-pV carriers (Cibula et al. 2011). Although a metaanalysis from 2013 did not produce a significant result, it did conclude that the influence of oral contraception on the risk of breast cancer in women with pV in *BRCA1/2* genes is comparable to that in women in the general population (Moorman et al. 2013). A more recent metaanalysis revealed inconsistent findings on the influence of oral contraceptives on the risk of breast cancer in women with pV in *BRCA1/2* genes, depending on the calculation model used (van Bommel et al. 2023). Taking into account 11 studies and **7525** *BRCA1/2*-pV carriers, a significant increase in the risk of breast cancer was found (HR 1.55; 95% CI 1.36–1.82). The largest and only study to date with a prospective and retrospective study component of the International *BRCA1/2* Carrier Cohort Study (IBCCS consortium), was included in this metaanalysis (Schrijver et al. 2018). Furthermore, 6 studies with a total of **9106** *BRCA1/2*-pV carriers were considered, for which there was no significant association (OR 1.06; 95%CI 0.90–1.25) (van Bommel et al. 2023).

In the retrospective part of the study by Schrijver et al., a total of **5705** carriers of pV in *BRCA1* and 3,521 carriers of pV in *BRCA2* were found to have an increased risk of breast cancer as a result of taking oral contraceptives (BRCA1: HR 1.39; 95% CI 1.23–1.58; BRCA2: HR 1.52; 95% CI 1.28–1.81) (Schrijver et al. 2018). In the prospective part of the study, **2276** and **1610** *BRCA1/2*-pV carriers were included. No association was shown between the use of oral contraception and the risk of breast cancer in *BRCA1/2*-pV carriers (HR 1.08; 95% CI 0.75–1.56 and 1.75; 95%CI 1.03–2.97) (Schrijver et al. 2018). The combined analysis of retrospective and prospective data showed no increased risk due to past use of hormonal contraceptives for middle-aged women (40–50 years). The increased breast cancer risk after long-term use, especially before the first child that was observed in the retrospective results were not supported by the prospective analyses, neither for *BRCA1*-pV carriers nor for those with pV in the *BRCA2* gene*.* Younger women were underrepresented in this study. Whether the differences between the retrospective and prospective results are due to a survival bias in the retrospective arm or whether there is an actual correlation cannot be clarified at present. The effect of progestin-only contraception was also not investigated in this study.

In a recent modeling study, the aim was to facilitate decision-making on the use of combined oral contraceptives in *BRCA1/2*-pV carriers. The risks of breast, ovarian and endometrial cancer were investigated. The analyses showed that the use of oral contraceptives in *BRCA1/2*-pV carriers initially led to an increased risk of breast cancer and in the long term to a reduced risk of ovarian and endometrial cancer (Schrijver et al. 2022). Subanalyses assuming 10 years of oral contraceptive use by 10,000 carriers of pV in the *BRCA1* gene from the age of 20 resulted in the following estimate: 12 additional cases of triple negative breast cancer would have occurred by the age of 25, 86 by the age of30 and a total of 210 by the age of 35 (Schrijver et al. 2022).

#### Influence of the use of progestin-only contraceptives on breast cancer risk of non-diseased *BRCA1*/*2*-pV carriers

##### Oral contraceptives for endometriosis

The overall model calculation described above clearly shows the risk-increasing effect of oral contraception with a sharp increase in triple-negative breast cancer after the age of 30 (Schrijver et al. 2022). For women in special situations, such as endometriosis, who require treatment, which necessitate long-term progestin therapy, for example by the use of hormonal contraception, the option of a risk-reducing mastectomy could therefore take on greater significance. The following observations point out that progestin-only contraception is not a safe alternative in this situation:

The assessability of breast magnetic resonance imaging appears to be reduced in the second half of the cycle (Clendenen et al. 2013). At the same time, there is evidence of a higher mammographic density due to an endogenously or exogenously increased progesterone level (Gabrielson et al. 2020). It is known that mammographic density is the strongest non-genetic risk factor for breast cancer (McCormack and dos Santos Silva 2006; Lee et al. 2019). The use of progestin-only contraception could increase mammary gland density to varying degrees depending on the individual. This then represents an unfavourable prerequisite for participation in the intensified breast screening program. The influence of progestin-only contraception on the risk of breast cancer, as well as the relationship between mammographic density and breast cancer, are subject of current research with limited data and still contradictory study results.

##### Progestin-releasing intrauterine device

There are no data available for newer hormonal contraceptives, such as levornogestrel-releasing intrauterine devices (IUDs), for *BRCA1/2*-pV carriers. These should therefore only be prescribed after a risk–benefit assessment and strict indication. As mentioned above, an increased risk of breast cancer has been described for the general population after insertion of a progestin-releasing IUD (RR 1.21; 95% CI 1.11–1.33) (Mørch et al. 2018). An increase in breast cancer risk in *BRCA1/2*-pV carriers can therefore not be ruled out.

#### Summary

An increase in breast cancer risk due to hormonal contraception in *BRCA1/2*-pV carriers cannot be ruled out based on the current data. Available data is insufficient, particularly in the case of early initiation. Therefore, *BRCA1/2*-pV carriers should only use hormonal contraceptives after careful consideration and for as short a time as possible (< 5 years). Alternative contraceptive methods should be used from around the age of 30, when the underlying risk of breast cancer increases. Based on the current data, hormonal contraceptives can be used in adolescence and early adulthood, when a safe contraceptive method has the highest priority and the fewest alternatives exist. Participation in prospective registry studies is recommended.

### Influence of the use of hormonal contraceptives on breast cancer risk of non-diseased carriers of pV in other risk genes for breast and/or ovarian cancer

Currently, no studies exist regarding the influence of hormonal contraceptives on breast cancer risk in carriers of pV in breast and/or ovarian cancer genes other than *BRCA1* and *BRCA2*.

### Influence of the use of hormonal contraceptives on cancer risk of women in the general population after diagnosis of breast cancer

The available data on the use of hormonal contraceptives and their influence on the risk of local recurrence or distant metastases after breast cancer in the general population is limited. A Cochrane metaanalysis containing 5 randomized controlled trials with 543 breast cancer patients, in which use of a progestin-releasing intrauterine device was examined with simultaneous antihormonal therapy with tamoxifen, showed no increase in the risk of recurrence (Dominick et al. 2015). According to the current S3 guideline on hormonal contraception, an increased risk of recurrence cannot be ruled out due to the low number of cases (S3-Guideline “Hormonal Contraception”). Hormonal contraceptives should therefore not be used. This includes the use of progestin-releasing intrauterine devices.

The German S3-guideline on “Early detection, diagnosis, treatment and follow-up of breast cancer” recommends carefully weighing the risks of hormonal contraception when pregnancy prevention is indicated (S3-Guideline “Screening, Diagnosis, Treatment and Follow-up of Breast Cancer”).

### Influence of the use of hormonal contraceptives on cancer risk of *BRCA1/2*-pV carriers or carriers with pV in other breast and/or ovarian cancer risk genes after diagnosis of breast cancer

No studies are available on the influence of hormonal contraceptives on the risk of local recurrence or distant metastases for breast cancer in *BRCA1/2*-pV carriers or carriers with pV in breast and/or ovarian cancer genes other than *BRCA1* and *BRCA2*. The approach should be the same as for women with breast cancer from the general population.

## Hormone replacement therapy (HRT) after risk-reducing salpingo-oophorectomy (RRSO)

Does hormone replacement therapy (HRT) after risk-reducing salpingo-oophorectomy (RRSO) influence breast cancer risk of non-diseased *BRCA1/2*-pV carriers or carriers of pV in genes of Lynch syndrome?

Statements:

- Data on substitution of estrogens (ET), if necessary in combination with progestins (EPT), and breast cancer risk in *BRCA1/2*-pV carriers after RRSO is limited. Based on these, there appears to be at least no strong risk-increasing effect.
- There are no data available on HRT and risk of breast cancer in carriers of pV in the Lynch syndrome genes after RRSO.

Recommendations:

- Premenopausal *BRCA1/2*-pV carriers should be offered HRT to prevent the negative consequences of estrogen deficiency.
- HRT should be carried out until the natural menopausal age.

### Recommendations for HRT in the general population

There are no studies that have investigated the effect of HRT on the risk of breast cancer in women with premature ovarian insufficiency (POI) in the general population. For the general population, hormonal treatment is recommended for POI, i.e. loss of ovarian function before the age of 40, provided there are no contraindications (S3-Guideline “Peri- and Postmenopause – Diagnosis and Interventions). This should be carried out until natural menopausal age is reached. This recommendation also applies to iatrogenically induced POI, for example after bilateral salpingo-oophorectomy.

### Risks of premature menopause without HRT

For premenopausal women in the general population, a significantly increased cardiovascular risk as well as an increased overall mortality was observed after bilateral adnectomy before the age of 45 in addition to cognitive impairment (Faubion et al. 2015; Georgakis et al. 2019). An analysis of the Nurses’ Health Study by Parker et al. revealed a significantly increased risk of coronary heart disease (hazard ratio (HR) 1.17; 95% CI 1.02–1.35) and increased all-cause mortality (HR 1.12; 95% CI 1.03–1.21) if HRT was not used (Parker et al. 2009). The increased overall mortality after adnectomy before the age of 45 and without ET was confirmed in further studies (Rocca et al. 2006; Parker et al. 2013). In a recent systematic review with metaanalysis, data from 20 cohort studies published between 1998 and 2022 were analyzed (Liu et al. 2023). In total, data from 921,517 women were included. A significantly higher risk of type II diabetes mellitus, hyperlipidemia, coronary heart disease, stroke and total cardiovascular events was shown for women who entered menopause before the age of 45 (Liu et al. 2023).

In addition to the increased cardiovascular risk due to premature onset of menopause, the higher risk of osteoporosis must also be mentioned. Both a significantly lower bone density and an increased fracture risk are described in women with POI (Vega et al. 1994; van der Klift et al. 2004; Faubion et al. 2015). Estrogen substitution reduces the fracture risk.

These negative effects of RRSO have also been shown for women with pV in *BRCA1/2* genes. A systematic review by Vermeulen et al. showed comparable effects on bone and cardiovascular system after RRSO in women with pV in *BRCA1*/2 genes as in women in the general population (Vermeulen et al. 2017). The available studies provide evidence that the described negative effects after RRSO, and in particular the extent of osteopenia, are more pronounced than after a natural premature onset of menopause (Vermeulen et al. 2017; Gaba and Manchanda 2020). Osteopenia and osteoporosis are observed less frequently after estrogen substitution in carriers of pV in *BRCA1/2* genes than without (Challberg et al. 2011).

In addition to the mentioned effects, RRSO also leads to menopausal symptoms and sexual dysfunction, which can limit quality of life. These impairments can be reduced by estrogen substitution (Vermeulen et al. 2017).

### Influence of HRT after risk-reducing salpingo-oophorectomy (RRSO) on breast *cancer* risk of non-diseased *BRCA1/2*-pV carriers

There are only a few studies that have investigated the risk of breast cancer by HRT in non-diseased *BRCA1/2*-pV carriers after RRSO (Huber et al. 2021). Mostly, no adverse effect was found, although different dosages and preparations were not investigated.

In a prospective cohort study from 2005, the influence of short-term HRT after RRSO, on the risk of breast cancer was investigated (Rebbeck et al. 2005). 462 carriers of pV in *BRCA1*/2 genes were included. At a postoperative follow-up period of 3.6 years, HRT was not associated with a significant increase in breast cancer risk. In this cohort, 139 women underwent RRSO before the age of 50, with 64% of the women (n = 89) receiving HRT. Also in this subgroup, HRT had no significant influence on breast cancer risk after RRSO (HR 1.35; 95% CI 0.16–11.58). Overall, reduction of breast cancer risk observed with adnectomy was not reversed by HRT (Rebbeck et al. 2005). However, the significance of the study is limited by the low number of cases and the limited details recorded on the type and duration of HRT and the timing of RRSO.

Another prospective cohort study by Kotsopoulos et al. from 2018 included 872 *BRCA1/2*-pV carriers (Kotsopoulos et al. 2018). Also in this study, no association of HRT and risk of breast cancer was observed (HR 0.97; 95% CI 0.62–1.52, p = 0.89). The mean duration of use was 3.9 years with a follow-up period of 7.6 years. The subgroup analyses of ET and EPT also showed no increased risk. However, the small number of cases in individual subgroups limits clinical significance.

A metaanalysis from 2018, which was based on the last two mentioned studies by Rebbeck et al. 2005 and Kotsopoulos et al. 2018 and included a total of 1100 *BRCA1/2*-pV carriers, showed no association between HRT and the risk of breast cancer after RRSO (HR 1.01; 95% CI 0.16–1.54) (Marchetti et al. 2018).

A more recent retrospective cohort study by Michaelson-Cohen investigated the risk of breast cancer after RRSO in 306 non-diseased *BRCA1/2*-pV carriers (Michaelson-Cohen et al. 2021). Of these, 150 women received HRT (Michaelson-Cohen et al. 2021). In 156 women no HRT was administered. This analysis showed no significantly increased risk of breast cancer due to HRT after RRSO in *BRCA1/2*-pV carriers (Michaelson-Cohen et al. 2021). The authors carried out an age-dependent subgroup analysis. This did not reveal an increased risk of breast cancer for women under 45 years of age after RRSO that received HRT. However, they found an increased breast cancer risk in women aged 45 years and older who received HRT after RRSO (odds ratio (OR) 3.43; 95% CI, 1.2–9.8). It should be noted, that the number of breast cancer cases is low with a total of 36 cases (20 cases in the group with HRT and 16 cases in the group without hormonal therapy).

### Influence of HRT after risk-reducing salpingo-oophorectomy (RRSO) on breast cancer risk of non-diseased carriers of pV in other risk genes for breast and ovarian cancer

There are currently no studies that have investigated the influence of HRT after RRSO, on breast cancer risk in premenopausal carriers of pV in other risk genes for breast and ovarian cancer. If the risk of ovarian or tubal carcinoma is increased, the disease risks are lower compared to those in the presence of pV in the *BRCA1* and *BRCA2* genes and RRSO is generally not indicated until the onset of menopause.

After RRSO in premenopause, the negative effects of premature onset of menopause on bone health and the cardiovascular system must also be considered in these women. Therefore, after RRSO premenopausal carriers of pV in other risk genes for breast and ovarian cancer should also be offered HRT to prevent negative consequences of estrogen deficiency. HRT should be carried out until the natural age of menopause.

### Influence of HRT after risk-reducing salpingo-oophorectomy (RRSO) on breast cancer risk of non-diseased carriers of pV in genes of Lynch-syndrome

Breast cancer risk is not increased to a clinically relevant degree in carriers of pV in the Lynch genes compared to the general population (Dominguez-Valentin et al. 2020). There are currently no studies that have investigated the influence of HRT after risk-reducing salpingo-oophorectomy (RRSO) on breast cancer risk in premenopausal carriers of pV in genes of Lynch-syndrome. However, negative effects of a premature onset of menopause on bone health and the cardiovascular system must also be taken into account in these women.

Therefore, premenopausal carriers of pV in genes of Lynch-syndrome should also be offered HRT after RRSO, to prevent negative consequences of estrogen deficiency. HRT should be carried out until the natural age of menopause.

### Influence of HRT after risk-reducing salpingo-oophorectomy (RRSO) on cancer risk of premenopausal *BRCA1/2*-pV carriers or in carriers of pV in other breast and/or ovarian cancer genes or genes of the Lynch-syndrome after diagnosis of breast cancer

Based on available data regarding women with sporadic breast cancer, it can be assumed that HRT can increase risk of breast cancer recurrence (S3-Guideline “Peri- and Postmenopause – Diagnosis and Interventions). According to the S3-guidelines on “Early detection, diagnosis, treatment and aftercare of breast cancer” and “Peri- and postmenopause”, HRT should not be carried out in women after breast cancer. In individual cases, it may be considered after failure of non-hormonal therapies and if quality of life is significantly impaired (S3-Guideline “Screening, Diagnosis, Treatment and Follow-up of Breast Cancer”; S3-Guideline “Peri- and Postmenopause – Diagnosis and Interventions).

There are no studies on risk of HRT after RRSO on breast cancer recurrence in premenopausal *BRCA1/2*-pV carriers or of carriers of pV in other breast and/or ovarian cancer genes or genes of the Lynch-syndrome. However, it can be assumed that the influence of HRT on the risk of recurrence after breast cancer treatment in this cohort does not differ significantly from that in women with sporadic breast cancer. For this reason, HRT should not be carried out after RRSO in premenopausal *BRCA1/2*-pV carriers or of carriers of pV other breast and/or ovarian cancer genes or genes of the Lynch-syndrome after diagnosis of breast cancer. In individual cases, it may be considered after failure of nonhormonal therapies and if quality of life is significantly impaired.

### Summary

Premenopausal *BRCA1/2*-pV carriers or carriers of pV in other breast and/or ovarian cancer genes or genes of the Lynch-syndrome should be offered HRT after RRSO to counteract negative effects of estrogen deficiency. The fact that these recommendations are based on limited available data should be communicated to patients. Due to the possible increase of breast cancer risk, HRT should be discontinued once natural age of menopause has been reached.

Following treatment for early breast cancer, HRT is generally contraindicated. Use of HRT should therefore be discussed critically in these patients. Age, prognosis of the disease, adjuvant endocrine treatment, menopausal symptoms and non-oncological risks must be taken into account.

## Hormone replacement therapy (HRT) without risk-reducing salpingo-oophorectomy (RRSO)

Does hormone replacement therapy (HRT) influence breast and ovarian cancer risk of non-diseased *BRCA1/2*-pV carriers without risk-reducing salpingo-oophorectomy (RRSO)?

Statements:

- Data on HRT and breast or ovarian cancer risk in *BRCA1/2*-pV carriers without RRSO is limited.
- Based on the current data an increase in risk of breast and ovarian cancer as a result of HRT cannot be ruled out.

Recommendations:

- For *BRCA1/2*-pV carriers without RRSO, HRT can be considered for severe menopausal symptoms after failure of non-hormonal treatment options.
- Adequate information on the weak evidence and possible increase in cancer risk must be communicated.

### Influence of HRT on breast cancer risk in women without previous cancer

#### Influence of HRT on breast cancer risk in women without previous cancer in the general population

Studies on the influence of HRT on breast cancer risk in women in the general population show little or no increase in risk (S3-Guideline “Peri- and Postmenopause—Diagnosis and Interventions). The possible increase in risk depends on the type and duration of HRT and decreases after discontinuation (S3-Guideline “Peri- and Postmenopause—Diagnosis and Interventions).

Relative risk (RR) of EPT is 1.26 in the large randomized, controlled trials (RCT) of the “Women's Health Initiative (WHI)”. This corresponds to 8 additional invasive breast cancers per 10,000 women per year of use (S3-Guideline “Peri- and Postmenopause—Diagnosis and Interventions; Prentice et al. 2009; Manson et al. 2013). However, in this trial, the risk increase only became apparent after 5 or more years of EPT use (S3-Guideline “Peri- and Postmenopause – Diagnosis and Interventions). Current re-analyses of data from the WHI trials still show an increased breast cancer risk following the use of EPT (Chlebowski et al. 2020; Prentice et al. 2021). A metaanalysis by the “Collaborative Group on Hormonal Factors in Breast Cancer” from 2019 analyzed all prospective studies available between 1992 and 2018 on the type and timing of HRT (Collaborative Group on Hormonal Factors in Breast Cancer 2019). During the prospective follow-up, breast cancer occurred in 108,647 postmenopausal women, of whom 51% had used HRT. For women currently using EPT, RR during the first four years of use was 1.6 (95% CI 1.52–1.69). EPT use for 5 or more years increased the RR to 2.08 (95% CI 2.02–2.15). Cohort studies provide evidence for a higher risk increase with continuous combined EPT than with sequential EPT (S3-Guideline “Peri- and Postmenopause – Diagnosis and Interventions). RCTs and observational studies have shown that although breast cancer risk is increased by current use of EPT, it decreases after discontinuation of therapy and differs no longer from that of non-users after a few years (S3-Guideline “Peri- and Postmenopause—Diagnosis and Interventions). Furthermore, timing in relation to menopause has an impact on breast cancer risk. Data from the randomized controlled WHI study and several larger observational studies show a higher risk of breast cancer in women who used EPT at or shortly after menopausal age compared to those who were already 5 or more years postmenopausal (S3-Guideline “Peri- and Postmenopause—Diagnosis and Interventions).

In contrast to numerous observational studies, the WHI studies showed reduced breast cancer risk following estrogen therapy (ET) (Anderson et al. 2004). Recent re-analyses of the WHI data still found a reduced risk of breast cancer and coronary heart disease with the use of conjugated estrogens (Chlebowski et al. 2020; Prentice et al. 2021). Three other randomized controlled trials, which had lower case numbers and follow-up durations, found no significant difference in breast cancer risk between ET of different formulations and placebo users (Hodis et al. 2001; Viscoli et al. 2001; Cherry et al. 2002). Observational studies have shown a lower increase of breast cancer risk following use of ET compared to EPT (Colditz et al. 1992; Willis et al. 1996; Sourander et al. 1998; Lando et al. 1999; Schairer et al. 2000; Beral 2003; Bakken et al. 2004, 2011; Stahlberg et al. 2004; Tjønneland et al. 2004; Fournier et al. 2005, 2008; Lund et al. 2007; Saxena et al. 2010). The metaanalysis of the “Collaborative Group on Hormonal Factors in Breast Cancer” mentioned above also showed lower breast cancer risk after ET application than for EPT. However, breast cancer risk was still significantly increased by current ET use over a period of up to four years in this analysis (RR 1.17; 95% CI 1.10–1.26) (Collaborative Group on Hormonal Factors in Breast Cancer 2019). When ET was used for 5 or more years, RR increased to 1.33 (95% CI 1.28–1.37).

#### Influence of HRT on breast cancer risk in *BRCA1/2*-pV carriers without previous cancer who have not undergone RRSO

Data on the effects of HRT on breast cancer risk in *BRCA1/2*-pV carriers without RRSO are limited. There is only one case–control study from 2016, in which 864 *BRCA1*-pV carriers were included, of whom approx. 75% had not undergone adnectomy (Kotsopoulos et al. 2016). In this cohort, no association between use of HRT and breast cancer risk was observed (OR 0.80; 95% CI 0.55–1.16; p = 0.24). *BRCA2*-pV carriers were not examined in this study.

### Influence of HRT on ovarian cancer risk in women without previous cancer

#### Influence of HRT on ovarian cancer risk in women without previous cancer in the general population

Studies that have investigated the influence of HRT on ovarian cancer risk of women in the general population show a possible increased risk (S3-Guideline “Peri- and Postmenopause—Diagnosis and Interventions). The effect can already occur after less than five years of use and is reduced after discontinuation of therapy (S3-Guideline “Peri- and Postmenopause—Diagnosis and Interventions). In a metaanalysis by the “Collaborative Group on Epidemiological Studies of Ovarian Cancer”, 52 studies were reanalyzed and data from 21,488 postmenopausal women with ovarian cancer were included. The analysis showed an increase of ovarian cancer risk for current users even for periods of use of less than 5 years (RR 1.43; 95% CI 1.31–1.56, p < 0.001). Risk was increased both for ET and for EPT (Beral et al. 2015).

#### Influence of HRT on ovarian cancer risk in *BRCA1/2*-pV carriers without previous cancer who have not undergone RRSO

There are only one case–control study and one retrospective cohort study on the association between HRT and the risk of ovarian cancer in *BRCA1/2*-pV carriers (Huber et al. 2021). In the case–control study from 2006, 537 *BRCA1/2*-pV carriers were included. In this trial, no increase in ovarian cancer risk for *BRCA1/2*-pV carriers was observed following HRT (OR 0.93; 95% CI 0.56–1.56) (Kotsopoulos et al. 2006).

A retrospective cohort study from 2015 showed an increase of ovarian cancer risk following HRT both for pV-carriers in the *BRCA1* gene (OR 1.66; 95% CI 0.89–3.08; p < 0.001) and in the *BRCA2* gene (OR 3.04; 95% CI 1.19–7.8; p < 0.001) (Perri et al. 2015). However, the significance of this study is limited by its small size with only 105 participants and its low number of cases with 32 ovarian cancer cases in the HRT group and 73 cases among the controls. In addition, the main intention of the study was to investigate the relationship between cancer risk and fertility treatment. The association between the risk of ovarian cancer and duration and type of HRT was not investigated.

### Summary

Overall, data on the association between HRT and breast or ovarian cancer risk in *BRCA1/2*-pV carriers without RRSO is limited. In view of the influence of HRT on the risk of breast and ovarian cancer in the general population, an increase in risk following HRT for *BRCA1/2*-pV carriers cannot be ruled out based on the current data.

In the case of pronounced menopausal symptoms, HRT can be considered after failure of non-hormonal therapy options for *BRCA1/2*-pV carriers without RRSO. However, adequate information about the weak evidence and a possible increase in cancer risk must be provided.

### Influence of HRT on cancer risk in women with previous cancer

#### Influence of HRT on risk of recurrence in *BRCA1/2*-pV carriers or pV-carriers in other risk genes for breast and ovarian cancer without RRSO after diagnosis of breast cancer

Based on currently available data regarding women with sporadic breast cancer, it can be assumed that HRT can increase the risk of breast cancer recurrence or distant metastases (S3-Guideline “Peri- and Postmenopause—Diagnosis and Interventions). According to the S3-guidelines on “Early detection, diagnosis, treatment and follow-up of breast cancer” and “Peri- and postmenopause”, HRT should not be used in women after diagnosis of breast cancer. In individual cases, it may be considered after failure of non-hormonal therapies and if quality of life is significantly impaired (S3-Guideline “Screening, Diagnosis, Treatment and Follow-up of Breast Cancer”; S3-Guideline “Peri- and Postmenopause—Diagnosis and Interventions).

There are no studies on the influence of HRT on breast cancer recurrence in *BRCA1/2*-pV carriers without RRSO. However, it can be assumed that the influence of HRT after treatment of breast cancer in this cohort does not differ significantly from that in women with sporadic breast cancer. Therefore, HRT should not be carried out in *BRCA1/2*-pV carriers after diagnosis of breast cancer. In individual cases, it may be considered after failure of non-hormonal therapies and if quality of life is significantly impaired.

#### Influence of HRT on risk of recurrence in *BRCA1/2*-pV carriers or pV-carriers in other risk genes for breast and/or ovarian cancer without RRSO after diagnosis of ovarian cancer

There are only a few studies on the impact of HRT on the risk of recurrence of ovarian cancer (S3-Guideline “Peri- and Postmenopause—Diagnosis and Interventions). Therefore, no reliable statement can be made on the safety of HRT after treatment of sporadic ovarian cancer (S3-Guideline “Peri- and Postmenopause—Diagnosis and Interventions). According to the current S3-guidelines “Peri- and Postmenopause” and “Diagnosis, Therapy and Aftercare of Malignant Ovarian Tumors”, HRT can be carried out in women after treatment of ovarian cancer when appropriate information has been provided (S3-Guideline “Diagnosis, Treatment and Follow-up of malignant Ovarian Tumors”; S3-Guideline “Peri- and Postmenopause—Diagnosis and Interventions).

There are no available data dealing with the influence of HRT on the risk of recurrence after treatment of ovarian cancer in *BRCA1/2*-pV carriers. It cannot be assumed that the impact of HRT on the recurrence of ovarian cancer in *BRCA1/2*-pV carriers or pV-carriers in other risk genes for ovarian cancer differs significantly from that of women with sporadic ovarian cancer. Therefore, after ovarian cancer in premenopausal women, HRT can be carried out to prevent negative consequences of estrogen deficiency until the natural age of menopause, following the recommendations for patients without pV. Adequate information about the weak evidence and possible increase in the risk of relapse and the risk of secondary breast disease must be provided.

## Hormone replacement therapy (HRT) and endometrial cancer risk

Does hormone replacement therapy (HRT) influence endometrial *cancer* risk in BRCA1/2-pV carriers?

Statement:

- The effect of HRT on endometrial cancer risk of in *BRCA1/2*-pV carriers has not been sufficiently investigated.
  Recommendation:
- HRT after RRSO for *BRCA1/2*-pV carriers should be carried out in line with the recommendations for patients without pV.

### *BRCA1/2*-pV-carriers and endometrial cancer risk

Few studies provide evidence of an increased risk of endometrial cancer in *BRCA1/2*-pV carriers (Thompson and Easton 2002; Segev et al. 2013; Laitman et al. 2019). However, some studies showed clear limitations due to influencing factors such as the use of tamoxifen.

A prospective cohort study by Shu et al., in which **1083** *BRCA1/2*-pV carriers were included, observed an increased risk of high-grade serous endometrial cancer in carriers of pV in the *BRCA1* gene between the ages of 45 and 70 (Shu et al. 2016). Another study describes an association between limited DNA repair of homologous recombination and the occurrence of serous endometrial carcinomas, suggesting a *BRCA1*/2-associated tumor (Jonge et al. 2017).

Due to the low risk of disease and lack of data on mortality reduction, hysterectomy, for example as part of an RRSO, is currently only recommended for *BRCA1/2*-pV carriers if there are additional reasons.

### Influence of HRT on endometrial cancer risk in the general population

Studies on the influence of HRT on the risk of endometrial cancer in women in the general population show that estrogen therapy (ET) without additional use of progestin significantly increases endometrial cancer risk in non-hysterectomized women (S3-Guideline “Peri- and Postmenopause—Diagnosis and Interventions). Following use of EPT containing conjugated equine estrogens and medroxyprogesterone acetate, a reduced endometrial cancer risk was observed after an average duration of use of 5.6 years. Accordingly, continuous EPT for less than 5 years can be considered safe. Longer use can lead to an increased endometrial cancer risk. The long-term use of progesterone or dydrogesterone as part of continuous EPT can also increase endometrial cancer risk. Risk of endometrial cancer may be increased when using sequential EPT, depending on the duration, type and dose of the progestin. However, sequential combined HRT over a period of less than 5 years using a synthetic progestin is considered safe with regard to the risk of endometrial cancer, provided that the progestin is used for at least 10, preferably 14 days.

A systematic review from 2020 analyzed 31 publications with data from 21,306 women with endometrial cancer (Tempfer et al. 2020). A significantly reduced risk of endometrial cancer was found after the use of continuous combined HRT with synthetic progestins with HRs between 0.24 and 0.71 (Tempfer et al. 2020). The extent of the risk reduction depended on the duration of use. A significant increase in risk was found in users of sequential combined EPT in 6 of 12 studies with ORs and HRs between 1.38 and 4.35. Duration of monthly progestin use was a significant modulator of endometrial cancer risk (Tempfer et al. 2020).

### Influence of HRT on endometrial cancer risk in *BRCA1/2*-pV carriers

There is one case–control study that investigated the association between use of HRT and endometrial cancer risk in *BRCA1/2*-pV carriers (Segev et al. 2015). 83 endometrial cancer cases and 1027 controls were included and 20.5% of cases (n = 17) and 7.4% (n = 76) of controls had used tamoxifen. In this study cohort, no association between HRT and endometrial cancer risk was observed (OR 0.73; 95% CI 0.33–1.63; p = 0.44) (Segev et al. 2015). However, the trial is limited by a low number of HRT users with only 13 women with endometrial cancer and 157 women in the control group.

### Summary

The impact of HRT on endometrial cancer risk in *BRCA1/2*-pV carriers has not been sufficiently investigated. Nevertheless, HRT after RRSO can be used in *BRCA1/2*-pV carriers in line with the recommendations for patients without pV. ET is contraindicated in non-hysterectomized *BRCA1/2*-pV carriers. Continuous combined EPT for less than 5 years can be considered safe. The use of progesterone or dydrogesterone as part of continuous combined EPT may increase the risk of endometrial cancer. Furthermore, sequential combined EPT over a period of less than 5 years with the use of a synthetic progestin is considered safe with regard to the risk of endometrial cancer, provided that progestin is used for at least 10, preferably 14 days.

## Conclusion

This paper provides a comprehensive overview of the current data on the risk of endocrine interventions in women with a genetic predisposition for breast and gynecological malignancies. This topic is of high relevance in everyday clinical practice, as there is still considerable uncertainty in dealing with hormonal therapies in this context. Data on risk modulation by endocrine interventions in women with a genetic predisposition for breast and gynecological malignancies is limited. Ovarian hyperstimulation with clomiphene or gonadotropins probably does not increase breast cancer risk in *BRCA1*/2-pV carriers or pV-carriers in other risk genes for breast and ovarian cancer. Due to limited and low quality evidence, definitive conclusions on oncological safety cannot be reliably made. Current data does not provide clear evidence on the potential risk-increasing effect on ovarian cancer. Therefore, ovarian hyperstimulation for fertility treatment in *BRCA1/*2-pV carriers or pV-carriers in other risk genes for breast and ovarian cancer can be performed. However, information about the limited data on oncological safety should be provided. Oral contraceptives significantly reduce ovarian cancer risk in *BRCA1*/2-pV carriers or pV-carriers in other risk genes for breast and ovarian cancer. However, oral contraceptives should not be used to reduce ovarian cancer risk in *BRCA1*/2-pV carriers or pV-carriers in other risk genes for breast and ovarian cancer. There is evidence of an age-dependent, risk-increasing effect of oral contraceptives on breast cancer risk in these carriers. Therefore, oral contraceptives should be used cautiously and after careful consideration of age and other factors. Data on HRT and breast cancer risk in *BRCA1*/2-pV carriers after RRSO is limited. It does not appear to have a strong risk-increasing effect. Premenopausal *BRCA1*/2-pV carriers should be offered HRT after RRSO until the natural age of menopause to prevent negative effects of estrogen deficiency. An increased risk of breast and ovarian cancer due to HRT in *BRCA1*/2-pV carriers without RRSO cannot be excluded based on current data. Therefore, HRT can be considered for severe climacteric symptoms if non-hormonal treatments fail after adequate counseling on the weak evidence and potential increased cancer risk.

In order to better evaluate the effects of endocrine interventions in women with a genetic predisposition to breast cancer and gynecological malignancies, it is essential to document them in prospective registries, as the HerediCaRe study does. This will add evidence for counseling women regarding their individual risk by endocrine interventions in the future.

## Data Availability

The datasets generated during and/or analysed during the current study are available from the corresponding author on reasonable request. No datasets were generated or analysed during the current study.
